# **Changes in Preterm and Low Birthweight Birth Rate During the COVID-19 Lockdown at Two San Francisco Hospitals**

**DOI:** 10.1007/s44197-025-00415-9

**Published:** 2025-05-19

**Authors:** Anoushka Kathiravan, Zoe R. Schauer, Janet M. Wojcicki

**Affiliations:** 1 Pediatric Gastroenterology, Hepatology and Nutrition, University of California, San Francisco, CA USA; 2 Epidemiology and Biostatistics, University of California, San Francisco, CA USA

**Keywords:** COVID-19, Lockdown, Preterm birth, Low birth weight, San Francisco

## Abstract

**Objective:**

The COVID-19 lockdown impacted interactions with the health care system and societal stress levels. Previous US-based studies suggest that pandemic lockdowns may have lowered preterm birth rates although there are mixed findings from different settings. We evaluated the impact of COVID-19 on preterm birth and low birthweight rates at two San Francisco hospitals.

**Methods:**

We compared rates of preterm birth (< 37 weeks) and low birthweight (< 2500 g) in San Francisco at a safety net hospital and an academic medical center during two time periods early in the COVID-19 pandemic compared with the same months from the prior year: from March to May 2019 and 2020 and August to December 2019 and 2020. We calculated crude rates for preterm birth and low birth weight as well as compared maternal and infant birth demographics and health characteristics during these same time periods using descriptive statistics. Secondly using a cross-sectional study design, we used logistic and linear regression models to evaluate risk for preterm birth, gestational age at birth, and low birthweight adjusting for confounders comparing the lockdown period with the pre-COVID year. All analyses were conducted using Stata 15.0.

**Results:**

From August to December 2019 to 2020, the preterm birth rate decreased from 13.20 to 7.96% in the combined hospital data (*p* < 0.01), and the low birthweight rate decreased from 11.33 to 9.70% during the same time period (*p* = 0.13). We did not find a comparable reduction from March to May 2019 to 2020. Maternal age at delivery was significantly younger during the lockdown period, August to December, than in the prior year (36.29 ± 5.69 versus 37.15 ± 5.68 years *p* < 0.01) and parity was greater (0.83 ± 1.15 versus 0.74 ± 1.04, *p* = 0.03) but there were no other significant differences in race or ethnicity, infant sex or type of delivery (vaginal versus Cesarean section) from 2019 to 2020. In a logistic and linear regression model adjusting for maternal age and infant sex and other confounders, the lockdown period from August to December was protective against preterm birth (OR 0.65, 95%CI 0.51–0.82) and associated with overall longer gestational duration (Coeff 0.23, 95%CI 0.07–0.39). The August to December lockdown period was also associated with greater birthweight (Coeff 43.76, 95%CI 2.19–85.34).

**Conclusions:**

In San Francisco, COVID-19 lockdowns lowered the preterm birth and increased gestational duration in infants comparing 2019 with 2020 for August to December. The reduced in preterm birth rates may be related to the overall strict lockdown measures that San Francisco implemented compared with other US cities.

**What this Study Adds to the Clinical Work?:**

This study suggests that COVID-19 lockdowns lowered the preterm birth rate in August -December 2020 compared with the same months in 2019 in two hospitals in San Francisco. San Francisco had strict lockdowns compared with other areas of the country and fewer deaths. The stay-at-home measures could possibly have reduced stress for pregnant women or had other positive benefits that reduced the preterm birth rate.

## Background

COVID-19, the global pandemic set off waves of international quarantines that severely impacted daily life [1]. People were forced to stay indoors, work from home, and limit social exposure. The extent of the quarantine’s effect was vast, yet long-term effects have not been elucidated. During the acute lockdown periods, routine access to hospitals and clinics was restricted, and associated psychological stress and/or routine changes in practices may have had varying impacts on a wide range of health conditions, including preterm birth (< 37 weeks gestation) and/or low birthweight (< 2500 g) [2].

Previous studies including meta-analyses and large prospective studies have found that maternal mental health including anxiety and/or depression in pregnancy may increase risk for preterm birth outcomes [3, 4]. The effect of COVID-19 on maternal mental health has been inconclusive and could have indirectly impacted the preterm birth rate via shifts in maternal mental health. Stress from isolation and irregular routines may have increased depression and anxiety in pregnant women during the pandemic [5]. However, limiting stress from work, travel, and day-to-day activities may also have benefited mothers’ quality of life. A comprehensive U.S. study found a decrease in the number of preterm births using an interrupted time-series approach to determine if there was a significant difference between the expected number of preterm births in 2020 and the actual rate [6]. The impact of the COVID-19 pandemic on maternal stress and mental health and subsequent risk for preterm birth was likely not uniform and differed by time-period (months of 2020 versus 2021 and later when the COVID-19 vaccine was available) and regional location.

Regional data suggests a heterogenous impact on the preterm birth rate with no impact in the urban area of Philadelphia, while New York City had a reduction [7, 8]. A statewide study in California found that there was no change overall in the preterm birth rate from pre-COVID years to 2020 [9]; however, as regional and county-specific guidelines were not uniform in California, it is possible that there was heterogeneity in preterm birth rates. San Francisco County had one of the most extensive lockdowns of all California counties [10].

The COVID-19 pandemic also impacted access to clinical and hospital care. Overall, a greater number of antenatal care visits are associated with reduced risk for preterm birth in a dose dependent manner including reduced rates in high-risk groups [11–13]. Studies evaluating the COVID-19 epidemic impact on access to prenatal care suggest that prenatal care visits declined during the lockdown period across all racial and ethnic groups with in-person visits potentially being the most impacted [14, 15].

We conducted a study comparing preterm birth rates and other birth outcomes including gestational age at birth, birthweight and low birthweight between 2019 (pre-pandemic) and 2020 (during the months of the more severe lockdowns, March-December) at two hospitals in San Francisco. San Francisco was an urban area in the United States with one of the most severe lockdowns but also reduced deaths from COVID-19 compared with other urban areas.

## Methods

### Variables

#### Study Population and Outcome

This study was conducted using data from the University of California, San Francisco Benioff (UCSF) and Zuckerberg San Francisco General (ZSFG) hospitals to assess for differences in preterm birth rate, very preterm (< 32 weeks gestation), gestational age (continuous), and low birth weight during the COVID-19 epidemic compared with the year prior to COVID-19. Difference in preterm birth rate was the primary outcome of interest and the others were secondary outcomes. Secondary, we assessed the demographics and birth characteristics (weight and gestational age) of preterm and low birthweight infants by birth year. As we compared the rate between two years, we conducted primarily descriptive analyses. Secondary, we analyzed the data cross-sectionally to assess risk factors for preterm birth, gestational age and low birth weight including year of birth. UCSF is a tertiary medical center and ZSFG is a public, safety net hospital serving the San Francisco Bay Area.

#### Study Data Collection

All births during the study time period were included in the database of births. For multiples, we only included the birth characteristics of the first listed multiple and did not include the additional infants. Gestational age at birth was calculated based on ultrasound dating or last menstrual period with similar protocols used at both hospitals. Standard digital scales were used to calculate birthweights also with similar protocols at both hospitals and gestational age was based on early transabdominal or transvaginal ultrasound dating.

#### Exposures

Our main exposure of interest was time as indicated by month and year as the data was compiled in a de-identified way as described below and the study received exempt status from the UCSF IRB. Births were combined for UCSF and ZSFG for August through December 2019 and compared with August through December of 2020 (the pre and post COVID period respectively). We also had an earlier time period available for UCSF only so also compared March through May 2019 (pre-COVID) with March through May 2020 (COVID) as a secondary analysis as medical records were not in an electronic format at ZSFG in this earlier time period. These earlier months of the pandemic (March to May) had a stricter lockdown with more restrictions on movement and more closures of businesses than the later months (August through December) in San Francisco. As such, we were able to evaluate these two different exposures (later lockdown (August through December 2020)) versus an earlier lockdown (March through May 2020) on risk for preterm birth, with the earlier time period limited by a smaller sample size. Furthermore, the earlier time period included women who experienced the first and second trimesters of gestation outside of a COVID-19 time-period.

#### Covariates

Maternal covariates evaluated included self-reported race (Black, Asian, White, Native American), self-reported ethnicity (Hispanic versus non-Hispanic), health characteristics (vaginal versus Cesarean section birth, previous history of preterm birth (self-reported) and parity (self-reported), and maternal age (years)) and infant covariates included sex (male versus female). Both hospitals included only self-reported race and ethnicity covariates.

#### Study Design and Statistics

UCSF’s Clinical and Translational Science Institute (CTSI) extracted and compiled the data from UCSF’s electronic record system (EPIC systems). Descriptive statistics were used to describe findings. Continuous data are reported as means ± standard deviation (SD), and categorical data are reported as percentages (%). All covariates were assessed for normality using Shapiro-Wilk tests. Non-parametric tests were used for data that were not normally distributed, including the Mann-Whitney U and chi-square tests. Gestational age and birthweight were not normally distributed and as such we used non-parametric tests.

As primary outcome of interest was the preterm birth rate in 2019 and 2020 with secondary outcomes including gestational age, birthweight and low birthweight. We calculated crude rates for preterm birth and low birthweight and mean gestational age and birthweight by year. Newborns that did not have a gestational age or birthweight were excluded from the analysis.

We also assessed associations between our outcomes of interest (preterm birth, low birthweight, infant gestational age, and birthweight) and maternal predictors including racial background, ethnicity (Hispanic or not Hispanic), previous history of preterm birth (yes or no), parity (number of previous live births) and birth type (Cesarean section or vaginal birth), and infant specifics (e.g. sex, male versus female) using cross-sectional data analysis approaches including the Mann-Whitney U test and the chi-squared test. We evaluated these maternal and infant factors in relation to births in the select months of 2019 versus 2020 (as described above) to assess if there were any differences in the maternal and infant demographic or health characteristics between the years that could explain differences between the two years in terms of preterm birth rate, low birthweight rate, gestational duration or birthweight means. A p-value less than 0.05 is considered statistically significant. All analyses were conducted using Stata 15.0.

So as to adjust for potential confounders and any biases related to confounding, we used logistic and linear regression models to evaluate independent predictors for preterm birth, low birth weight, gestational duration and birthweight adjusting for variables that were associated with our outcome in bivariate analysis, previous studies of preterm birth and possibly the time of delivery including maternal age at delivery (years), previous history of preterm birth and infant sex (male versus female), ethnicity (Hispanic versus non-Hispanic), race (Native-American/American-Indian, white, Asian, black or other/not answered). We also adjusted all analyses for hospital of birth as the lockdown may have had differential impacts on health seeking behaviors at the two hospitals that could have impacted preterm birth rate. The amount of missing data in the overall dataset was low (between 0.7 and 1.3%) with data only missing from birthweight, infant racial background and gender. A complete case analysis approach was used in the multivariable analysis. The study received exempt approval from the Institutional Review Board (IRB) of the University of California, San Francisco.

## Results

This study compares the gestational duration and birthweight of 1712 infants born in 2019 and 1482 infants born in 2020 at UCSF Benioff and ZSFG hospitals from August through December. Secondary, we assessed the gestational duration and birthweight of 582 infants born from March through May 2019 compared with 620 infants born in the same months in 2020 at UCSF Benioff hospital.

### Gestational Age, Preterm Birth, Birthweight and Low Birthweight by Birth Year (2019 Versus 2020)

In the combined hospital data, the mean gestational age of infants was shorter in 2019 than in 2020 although the results were not statistically significant. (38.56 ± 2.90 weeks versus 38.69 ± 2.77 weeks; *p* = 0.22; Table 1). This difference corresponds to approximately 1 day longer gestation. In the March-May data, it was longer in 2019 (38.79 ± 2.63 versus 38.60 ± 2.60 weeks; *p* = 0.04; Table 1).

**Table 1:** Maternal and infant characteristics in relation to birth year for UCSF and ZSFG hospitals

Maternal and Infant Variables		UCSF and ZSFG Births 2019* % (*N*/Total) or Mean ± SD	UCSF and ZSFG Births 2020* % (*N*/Total) or Mean ± SD	*p*-value	UCSF Births 2019** % (*N*/Total) or Mean ± SD	UCSF Births 2020** % (*N*/Total) or Mean ± SD	*p*-value
**Infant Specifics**							
Infant Gestational Age	Gestational age, weeks	38.56 ± 2.90	38.69 ± 2.77	0.22	38.79 ± 2.63	38.60 ± 2.60	**0.04**
	Preterm (< 37 weeks)	13.20 (226/1712)	7.96 (118/1482)	**< 0.01**	11.86 (69/582)	11.51 (71/617)	0.85
	Very Preterm (< 32 weeks)	3.33 (57/1712)	1.75 (26/1482)	**< 0.01**	2.58 (15/582)	3.24 (20/617)	0.50
Infant Birthweight	Birthweight, grams	3180.25 ± 649.28	3211.12 ± 626.55	0.13	3235.66 ± 621.49	3200.04 ± 640.04	0.36
	Low Birthweight, grams	1896.66 ± 572.97	1860.93 ± 588.36	0.43	1879.61 ± 589.44	1869.67 ± 564.12	0.84
	Low Birthweight%	11.33 (193/1703)	9.70 (151/1556)	0.13	8.67 (51/588)	10.32 (64/620)	0.33
Infant Sex	Female	48.50 (823/1697)	49.81 (769/1544)	0.46	50.51 (296/586)	50.57 (313/619)	0.99
	Male	51.50 (874/1697)	50.19 (775/1544)	0.46	48.95 (290/586)	49.43 (306/619)	0.99
**Maternal Specifics**							
Maternal Age, years		37.15 ± 5.68	36.29 ± 5.69	**< 0.01**	39.17 ± 5.01	38.40 ± 4.68	**< 0.01**
Maternal Ethnicity	Hispanic	27.01 (463/1714)	27.70(434/1567)	0.66	11.32 (67/582)	11.59 (72/621)	0.88
Maternal Race	Black	7.53 (129/1714)	7.72 (121/1567)	0.17	6.42 (38/592)	6.60 (41/621)	0.90
	Asian or Pacific Islander	20.60 (353/1714)	21.51 (337/1567)	0.17	27.36 (162/592)	24.64 (153/621)	0.28
	White	36.00 (617/1714)	38.16 (598/1567)	0.17	47.47 (281/592)	45.73 (284/621)	0.55
	Native American or Alaska Native	0.41 (7/1714)	0.77 (12/1567)	0.17	0.68 (4/592)	0.81 5/621)	0.79
Cesarean Section		20.89 (358/1714)	21.76 (341/1567)	0.54	23.99 (142/592)	20.13 (125/621)	0.11
Prior Preterm Birth		5.95 (102/1712)	6.06(95/1567)	0.89	4.90 (29/592)	6.44 (40/621)	0.24
Parity		0.74 ± 1.04	0.83 ± 1.15	**0.03**	0.62 ± 1.01	0.68 ± 0.94	**0.02**

*Births from August-December of 2019 and 2020

**Births from March-May of 2019 and 2020

^Numbers do not add up always due to missing data

In the combined hospital data, there was a significantly higher percentage of preterm infants born in 2019 than that of 2020 (13.20% versus 7.96% *p* < 0.01). The very preterm birth rate followed this pattern (3.33% versus 1.75% *p* < 0.01). There was no statistically significant difference in the rate of preterm birth in the earlier (March through May) hospital data from UCSF alone comparing 2019 and 2020.

In the combined hospital data, the average birthweight of infants born in 2019 was lower than that of infants born in 2020 but the results were not statistically significant ( 3180.25 ± 649.28 g versus 3211.12 ± 626.55 g; *p* = 0.13; Table 1). There was no difference in the low birthweight rate between 2019 and 2020 for the combined hospital sample or the UCSF only sample population (Table 1).

There were no significant differences in maternal or infant demographics or birth characteristics in relation to year of birth (2019 versus 2020) with the exception of maternal age and parity. Mothers with preterm births had a higher maternal birth age in 2019 versus 2020 for the combined and UCSF hospitals (37.15 ± 5.68 versus 36.29 ± 5.69 years, *p* < 0.01 and 39.17 ± 5.01 versus 38.40 ± 4.68 years, *p* < 0.01; Table 1). Maternal parity was also lower prior to COVID (2019) than in 2020 (0.62 ± 1.01 versus 0.68 ± 0.94, *p* = 0.02).

### Maternal and Infant Characteristics in Relation To Preterm Birth, Gestational Age, Low Birthweight and Birthweight by Birth Year (2019 Versus 2020)

We evaluated demographic and health characteristic differences between preterm birth, gestational age, birthweight and low birthweight comparing 2019 and 2020 (Table 2). For the combined hospital sample from August-December, there were differences between 2019 and 2020 in the mean gestational age of preterm infants by race. The gestational age in African-American preterm infants was shorter in 2020 than in 2019 (28.60 ± 6.22 versus 32.61 ± 4.95 weeks; Table 2; *p* < 0.01). Similarly, birthweight was lower for African-American preterm infants in 2020 compared with 2019 (1417.52 ± 755.60 versus 2142.21 ± 852.35 g (*p* = 0.02)). Maternal age was less in 2020 for the mothers of preterm infants and parity was higher (36.57 ± 6.13 versus 35.88 ± 6.31 years, *p* < 0.01) and 0.87 ± 1.18 versus 1.32 ± 1.79, *p* < 0.01.

**Table 2:** Maternal and infant characteristics for preterm and low birthweight infants at UCSF and ZSFG*^

Maternal and Infant Variables		Preterm Births 2019 % (*N*/Total) or Mean ± SD	Preterm Births 2020 % (*N*/Total) or Mean ± SD	*p*-value	Preterm Gestational Age (wks) 2019 Mean ± STD	Preterm Gestational Age (wks) 2020 Mean ± STD	*p*-value	Preterm Birthweight 2019 Mean ± STD	Preterm Birthweight 2020 Mean ± STD	*p*-value	Low Birth-weight 2019 % (*N*/Total) or Mean ± STD	Low Birth-weight 2020 % (*N*/Total) or Mean ± STD	*p*-value
Infant Sex	Female	50.94 (108/212)	42.65 (58/136)	0.13	33.58 ± 3.55	33.31 ± 2.89	0.10	2056.4 ± 632.50	2132.83 ± 703.67	0.29	9.16 (80/873)	9.96 (77/773)	0.58
	Male	49.06 (104/212)	57.35 (78/136)		33.58 ± 3.56	33.31 ± 2.89	0.10	2208.79 ± 684.54	2044.28± 678.11	0.07	13.08 (107/818)	7.93 (61/769)	**< 0.01**
Ethnicity	Hispanic	31.42 (71/226)	32.91 (52/158)	0.76	32.07 ± 4.84	32.87 ± 4.46	0.22	1967.73 ± 870.70	1381.26 ± 709.86	0.86	10.50 (48/457)	10.65 (46/432)	0.94
Race	Black	10.62 (24/225)	11.39 (18/158)	0.50	32.61 ± 4.95	28.60 ± 6.22	**< 0.01**	2142.21 ± 852.35	1417.52 ± 755.60	**0.02**	18.75 (24/3128)	19.33 (35/335)	0.91
	Asian/ Pacific Islander	18.14 (41/226)	16.46 (26/158)		34.42 ± 3.03	33.63 ± 3.41	0.30	2275.87 ± 522.10	2217.96± 610.61	0.69	10.51 (37/352)	10.45 (35/335)	0.97
	White	31.42 (71/226)	31.36 (37/158)		32.85 ± 4.47	33.60 ± 3.35	0.55	2060.54 ± 724.46	2161.84± 673.68	0.65	9.59 (59/615)	5.88 (35/595)	**0.02**
	Native American/ Alaska Native	0.44 (1/226)	2.53 (4/158)		31.29	31.50 ± 2.74		1659.86	1790.77 ± 848.67		14.29 (1/17)	33.33 (4/12)	0.36
Birth Type	Cesarean Section	37.61 (85/226)	37.34 (59/158)	0.96	33.71 ± 2.71	32.74 ± 3.32	0.08	2138.01 ± 686.23	2046.63 ± 678.77	0.50	18.72 (67/358)	16.18 (55/340)	0.38
Mom Age, years		36.57 ± 6.13	35.88 ± 6.31	**<0.001**							36.94 ± 6.36	35.56 ± 6.42	0.05
Previous preterm birth		15.93 (36/226)	18.99 (30/158)	0.43	32.08 ± 4.97	32.89 ± 4.27	0.49	1935.73 ± 964.89	2116.14 ± 587.45	0.77	30.69 (31/101)	26.09 (24/92)	0.48
Parity		0.87 ± 1.18	1.32 ± 1.79	**< 0.001**							0.78 ± 1.08	1.09 ± 1.60	**0.04**

*Births from August-December of 2019 and 2020

^Numbers do not add up always due to missing data

Comparing demographics and birth characteristics for low birthweight infants, there were fewer female low birthweight infants in 2020 than 2019 (7.93% (61/796) versus 13.08% (107/818), *p* < 0.01). There were also fewer white low birthweight infants in 2020 than in 2019 (5.88% (35/595) versus 9.59% (59/615), *p* = 0.02). Parity was also greater in 2020 than 2019 (1.09 ± 1.60 versus 0.78 ± 1.08. *p* = 0.04). There were no other significant demographic or maternal characteristic differences between the preterm birth or low birthweight infants between 2019 and 2020 (Table 2). We also did not find any other demographic or birth characteristic differences by gestational age or birthweight differences for preterm infants in 2019 versus 2020 (Table 2).

For the UCSF-only sample comparing the earlier COVID-19 time period March to May 2019 and 2020, we did not find any differences in demographics or maternal health characteristics of preterm or low birthweight infants or when we assessed these characteristics in relation to the gestational age or birthweight by preterm birth status (Table 3).

**Table 3:** Maternal and infant characteristics for preterm and low birthweight infants at UCSF Hospital*^

Maternal and Infant Variables		Preterm Births 2019% (*N*/Total) or Mean ± SD	Preterm Births 2020% (*N*/Total) or Mean ± SD	*p*-value	Preterm Gestational Age (wks) 2019 Mean ± STD	Preterm Gestational Age (wks) 2020 Mean ± STD	*p*-value	Preterm Birthweight 2019 Mean ± STD	Preterm Birthweight 2020 Mean ± STD	*p*-value	Low Birth-weight 2019 % (*N*/Total) or Mean ± STD	Low Birth-weight 2020 % (*N*/Total) or Mean ± STD	*p*-value
Infant Sex	Female	51.56 (33/64)	49.28 (34/69)	0.79	34.27 ± 2.32	33.34 ± 3.20	0.35	2303.33 ± 570.95	2133.72± 807.30	0.31	6.92 (20/289)	8.50 (26/306)	0.47
	Male	48.44 (31/64)	50.72 (35/69)		33.86 ± 2.75	33.27 ± 3.58	0.63	2391.83 ± 848.30	2211.72 ± 782.40	0.35	6.92 (20/289)	8.50 (26/306)	0.47
Ethnicity	Hispanic	18.84 (13/69)	18.31 (13/71)	0.94	33.23 ± 4.52	33.37 ± 2.55	0.50	2207.72 ± 1001.16	2121.76 ± 819.99	0.85	14.93 (10/67)	18.06 (13/72)	0.62
Race	Black	5.80 (4/69)	11.27 (8/71)	0.25	34.07 ± 1.55	34.07 ± 2.18	0.86	2430.69 ± 631.28	2327.21 ± 816.35	0.56	18.42 (7/38)	19.51 (8/41)	0.90
	Asian	24.64 (17/69)	21.13 (15/71)	0.62	33.97 ± 2.63	32.41 ± 4.74	0.25	2194.36 ± 589.97	1964.97 ± 706.14	0.34	8.70 (14/161)	9.21 (14/152)	0.87
	White	37.68 (26/69)	33.80 (24/71)	0.63	33.75 ± 3.38	33.80 ± 3.74	0.55	2614.47 ± 687.10	2453.30 ± 156.03	0.43	5.04 (14/278)	6.34 (18/284)	0.51
	Native American	1.45 (1/69)	0 (0/71)	0.31	35.29			N/A	N/A		N/A	N/A	
Birth Type	Cesarean Section	40.58 (28/69)	33.80 (24/71)	0.69	33.90 ± 2.34	32.54 ± 3.61	0.29	2212.39 ± 689.05	2019.77 ± 825.21	0.36	18.31 (26/142)	20.80 (26/125)	0.61
Mom Age, years		35.45 ± 5.57	34.10 ± 5.99	0.13							38.45 ± 6.01	37.78 ± 5.48	0.53
	Parity	0.86 ± 1.35	0.86 ± 1.09	0.44							0.82 ± 1.41	0.73 ± 1.01	0.69
	Prior preterm birth	1.86 ± 1.19	1.88 ± 1.36	0.65	31.27 ± 4.76	32.92 ± 3.01	0.40	1942.68 ± 702.17	20.92.28 ± 648.73	0.71	28.57 (8/28)	25.00 (10/40)	0.74

*Births from March-May of 2019 and 2020

^Numbers do not add up always due to missing variables

In multivariable models adjusting for maternal age at delivery, infant sex, ethnicity and race, previous preterm birth, parity and hospital of delivery, being born during the COVID-19 lockdown period (August-December 2020) was protective against preterm birth compared with the prior year (OR 0.65 95%CI 0.51–0.82; *p* < 0.01; Table 4). Previous history of preterm birth was also associated with increased risk (OR 3.75, 95%CI 2.61–5.40) while being born at ZSFG was protective against preterm birth (OR 0.44, 95%CI 0.32–0.62) (Table 4). In multivariable linear regression models adjusting for the same confounders, the lockdown period was associated with greater gestational age at birth (wks) (Coeff 0.23 95%CI 0.07–0.39) and higher birthweights (g) (Coeff 43.76, 95%CI 2.19–85.34) (results not shown). The lockdown period trended towards significance for reducing low birthweight infants but was not statistically significant (OR 0.79, 95%CI 0.62–1.01, *p* = 0.06).

**Table 4:** Multivariable model of risk factors for preterm birth at ZSFG and UCSF hospitals in 2020 versus 2019 (August-December)

	Unadjusted Odds Ratio (OR), 95%CI	*p*-value	Adjusted OR 95%CI	*p*-value
**Variables**				
Infant Sex (male versus female)	0.94 (0.75-1.17)	0.58	0.97 (0.77-1.22)	0.79
Lockdown Period (yes versus no)	0.74 (0.59-0.92)	<0.01	0.65 (0.51-0.82)	**<0.01**
Hispanic (yes versus no)	1.29 (1.03-1.63)	0.03	1.23 (0.88-1.73)	0.22
Mom’s age (years)	0.98 (0.97-1.00)	0.10	0.98 (0.95-1.00)	**0.047**
ZSFG (hospital)	0.73 (0.56-0.94)	0.02	0.44 (0.32-0.62)	**<0.01**
Race				
White	1.00		1.0	
Black	0.99 (0.72-1.36)	0.95	1.09 (0.79-1.52)	0.60
Asian/Pacific Islander	1.86 (1.27-2.72)	<0.01	1.46 (0.93-2.29)	0.10
Other	1.45 (1.13-1.88)	<0.01	1.37(0.97-1.93)	0.07
American Indian/	3.29 (1.16-9.29)	0.03	1.98 (0.60-6.47)	0.26
Alaska Native				
History of Previous Rreterm Birth	4.38(3.19-6.02)	<0.01	3.75 (2.61-5.40)	**<0.01**
Parity	1.24(1.14-1.35)	<0.01	1.10 (0.99-1.23)0.79	0.08

## Discussion

Our analysis found that from 2019 to 2020, there was a significant reduction in the number of preterm births in our two San Francisco hospitals during the later lockdown period August to December 2020 compared with these same months in 2019. There was also longer gestation in 2020 by approximately 1 day when the data was analyzed continuously, which could have overall clinical significance for neonatal health, however, our findings were not statistically significant. There was not a similar reduction comparing the earlier lockdown period (March to May of 2020 compared with the same months in 2019) although our numbers were smaller for this time period as we only had data from UCSF. We did not find a reduction in the rate of low birthweight infants from August to December of 2019 versus 2020.

These findings corroborate the trend observed across select areas of the United States of decreasing preterm birth rates during the COVID-19 lockdown [6] although our study is unique in that it compares the early and the later lowdown time period with similar months in 2019. Meanwhile, review articles universally emphasize an increase in stress and anxiety during the COVID-19 pandemic [16], and poor maternal mental health is associated with a higher risk of preterm birth [3]. Furthermore, day-to-day work and occupational stress contribute to a higher risk of preterm birth [2]. The pandemic resulted in many pregnant people staying at home, unable to work in person with the exception of essential workers. It is possible that stay at home mandates may have resulted in improvements in physical health for pregnant people in certain scenarios. San Francisco, in particular, had earlier remote work policies and local hospitals were less overwhelmed by COVID-19 compared with other urban areas, potentially, resulting in reduced anxiety for pregnant people compared with other urban areas [17].

Unilaterally, changes in hospital and clinic policies during the COVID-19 period also resulted in pregnant people needing to go to clinic visits alone which likely impacted health seeking behaviors that requires further study to better understand [18]. Furthermore, there were fewer in-person prenatal visits during 2020 which data suggest may result in greater risk for preterm birth, which is contrary to our findings and needs further study.

Interestingly, we did not find any differences in preterm birth rate comparing the early time period of COVID lockdown (March through May 2020) with the same months in 2019 versus the later analysis (August through December 2019 compared with 2020) although our numbers were smaller as we only had data from UCSF’s Benioff hospital. However, it is also possible that the earlier period of the lockdown may have been more associated with greater stress compared with later time periods. By May 18 2020, San Francisco county along with the other Bay Area counties planned a phase 2 re-opening [19]. However, this early time period also would have been less impacted by a reduction in in-person prenatal visits or changes in maternal mental health during the earlier gestational months as these pre-dated the beginning of the pandemic.

We did not find any reduction in low birthweight infants during the lockdown although there was an overall increase in birthweight. A systematic review of existing studies found that during the pandemic, there was a significant increase in the birthweight of infants and reduction in very low birthweight (< 1500 g) [20]. Another study in Ireland found a reduction in low birthweight and extremely low birthweight infants attributing the increase in infant birthweight to lower exposure to air pollutants, decreased work-related stress, and increased access to healthy nutrients in lockdown [21]. Alternatively, another earlier metanalysis did not find any impact on birthweight or gestational age suggesting possible heterogeneity based on regional differences in response [22]. It is possible that the benefits of the lockdown in the San Francisco area (e.g. reduced stress or less exposure to pollutants) may have outweighed any negative impacts (e.g. less access to prenatal care or hospital services).

We also found some changes to the demographic profile of preterm infants during the lockdown period versus prior to the lockdown. Black preterm infants, in particular, tended to have a shorter gestation and lower birthweight compared to prior to COVID-19 potentially related disparities or more stress in this population group. Further analyses could provide an explanation for this finding and connect it to widening health disparities after the onset of the pandemic including greater socioeconomic and racial and/or ethnic disparities in preterm birth [23–25]. Future studies are needed to better understand regional patterns in response to COVID-19 lockdowns and any differential response by race/ethnicity to lockdowns.

### Limitations

This study involved only two San Francisco hospitals, and as such had a small sample size and an even smaller sample size (only one hospital) to evaluate the impact of the early COVID-19 lockdown period. We also did not have data on additional risk factors for preterm birth including maternal mental health status, prenatal visit history or gestational hypertension or diabetes mellitus. However, our study provides further impetus to better assess the heterogeneous impact of the COVID-19 lockdown on health outcomes including preterm birth and low birthweight due to impact of different urban public health control measures during COVID-19. Future studies need to better assess the differential impact of region and city-specific measures on health changes during the COVID-19 period.

## Conclusion

Results from our study suggest a possible association between changes in lifestyle or maternal stress levels during the COVID-19 lockdown that could impact preterm birth levels. As we only found reductions in the later time period of lockdowns (August-December of 2020) compared with the earlier (March-May of 2020), it is possible that reduced stress in this period versus the earlier period may have positively impacted gestational duration and risk for preterm birth. As preterm birth may result in lifelong risk for additional morbidities and mortalities, effective interventions are needed including a better understanding of the casual pathways to preterm birth.

## Data Availability

Data can be made available on request to the corresponding author.
